# CRB-65 for Risk Stratification and Prediction of Prognosis in Pulmonary Embolism

**DOI:** 10.3390/jcm12041264

**Published:** 2023-02-05

**Authors:** Karsten Keller, Volker H. Schmitt, Ingo Sagoschen, Thomas Münzel, Christine Espinola-Klein, Lukas Hobohm

**Affiliations:** 1 Department of Cardiology, University Medical Center Mainz, Johannes Gutenberg-University Mainz, 55131 Mainz, Germany; 2 Center for Thrombosis and Hemostasis (CTH), University Medical Center Mainz, Johannes Gutenberg-University Mainz, 55131 Mainz, Germany; 3 Medical Clinic VII, Department of Sports Medicine, University Hospital Heidelberg, 69120 Heidelberg, Germany; 4 German Center for Cardiovascular Research (DZHK), Partner Site Rhine Main, 55131 Mainz, Germany

**Keywords:** pulmonary embolism, venous thromboembolism, pneumonia, mortality, risk stratification

## Abstract

Background: Pulmonary embolism (PE) is accompanied by high morbidity and mortality. The search for simple and easily assessable risk stratification scores with favourable effectiveness is still ongoing, and prognostic performance of the CRB-65 score in PE might promising. Methods: The German nationwide inpatient sample was used for this study. All patient cases of patients with PE in Germany 2005–2020 were included and stratified for CRB-65 risk class: low-risk group (CRB-65-score 0 points) vs. high-risk group (CRB-65-score ≥1 points). Results: Overall, 1,373,145 patient cases of patients with PE (76.6% aged ≥65 years, 47.0% females) were included. Among these, 1,051,244 patient cases (76.6%) were classified as high-risk according to CRB-65 score (≥1 points). The majority of high-risk patients according to CRB-65 score were females (55.8%). Additionally, high-risk patients according to CRB-65 score showed an aggravated comorbidity profile with increased Charlson comorbidity index (5.0 [IQR 4.0–7.0] vs. 2.0 [0.0–3.0], *p* < 0.001). In-hospital case fatality (19.0% vs. 3.4%, *p* < 0.001) and MACCE (22.4% vs. 5.1%, *p* < 0.001) occurred distinctly more often in PE patients of the high-risk group according to CRB-65 score (≥1 points) compared to the low-risk group (= 0 points). The CRB-65 high-risk class was independently associated with in-hospital death (OR 5.53 [95%CI 5.40–5.65], *p* < 0.001) as well as MACCE (OR 4.31 [95%CI 4.23–4.40], *p* < 0.001). Conclusions: Risk stratification with CRB-65 score was helpful for identifying PE patients being at higher risk of adverse in-hospital events. The high-risk class according to CRB-65 score (≥1 points) was independently associated with a 5.5-fold increased occurrence of in-hospital death.

## 1. Introduction

Pulmonary embolism (PE) is a cardiovascular emergency accompanied by high morbidity and mortality [1,2,3,4,5,6]. Mortality caused by PE is closely related to haemodynamic status and cardiac complications, including right ventricular dysfunction (RVD) and/or myocardial injury, as well as comorbidity profile [2,3,4,6,7,8,9,10,11,12,13]. The risk stratification of PE patients is crucial for choosing the appropriate therapeutic management [6]. According to the current ESC guideline for management of PE patients, initial risk stratification is based on clinical signs and symptoms as well as haemodynamic status, whereby haemodynamic instability indicates a high-risk status with substantially increased risk for early death [6]. In contrast, the majority of PE patients are haemodynamically stable, requiring further risk stratification of clinical, imaging, and laboratory indicators of RVD and PE severity, as well as the presence of a significant comorbidity profile and any other aggravating conditions that may adversely influence prognosis [6]. In the ESC risk stratification classification, the pulmonary embolism severity index (PESI) and the simplified pulmonary embolism severity index (sPESI) are implemented for the risk stratification approach to assess these mentioned significant comorbidity profiles and any other aggravating conditions [6]. The PESI compromises several parameters such as age, male sex, cancer, heart failure, chronic pulmonary diseases, tachycardia with heart rate ≥110 beats/min, systolic blood pressure <100 mmHg, respiratory rate >30 breaths/min and temperature <36.0 °C, altered mental status, and/or arterial oxyhaemoglobin saturation <90%, while sPESI is focused on the parameters of age >80 years, cancer, chronic heart failure or pulmonary disease, tachycardia with heart rate ≥110 beats/min, systolic blood pressure <100 mmHg, respiratory rate >30 breaths/min, and/or arterial oxyhaemoglobin saturation <90% [6,14,15,16,17].

Although several scores such as the PESI, sPESI, Bova score, FAST, and modified FAST-score, as well as others, are already in use for the prediction of PE prognosis [16,17,18,19,20,21,22,23,24,25,26,27,28], the search for simple and easily assessable scores with a favourable effectiveness is still ongoing [6,18]. The aforementioned scores are specialized for PE and lack generalization regarding prognosis evaluation of other pulmonary diseases [18]. In patients with pneumonia, CRB-65 is well validated and is widely used for severity assessment and the management of these patients [29,30,31,32,33,34] The major advantage of CRB-65 is that this score can easily be assessed and handled since the score is based exclusively on clinical, immediately assessable variables without technical expense [30,31,33,35] The criteria of CRB-65 comprise new-onset confusion, respiratory rate ≥30/min, systolic blood pressure <90 mmHg or diastolic blood pressure ≤60 mmHg, and age ≥65 years [29,31,33]. One small study demonstrated that the CRB-65 score might also be useful for risk stratification in patients with acute PE [18].

The objective of the present study is to investigate the use of CRB-65 for the prognosis prediction of patients with acute PE in a large nationwide inpatient sample.

## 2. Methods

The year 2004 was the start date from which all German hospitals have had to transfer their coded patient data, including diagnoses, (coexisting) conditions/comorbidities, surgeries, interventions, treatments, and procedures to the Institute for the Hospital Remuneration System and the Federal Statistical Office of Germany (Statistisches Bundesamt) in order to receive their remuneration for the provided and rendered services [3]. Patients’ diagnoses are coded according to ICD-10-GM (International Classification of Diseases, 10th Revision with German Modification), whereas surgical, diagnostic, and interventional procedures are coded according to OPS-codes (Operationen- und Prozedurenschlüssel) [3,36].

The information on all of these treatment data from the inpatient cases gathered by the Federal Statistical Office of Germany (Statistisches Bundesamt) is included in the German nationwide inpatient sample (diagnosis-related groups [DRG] statistic), which can be analysed and was used for this present study [3,36].

Our study analyses were computed and calculated by the Research Data Center (RDC) of the Federal Statistical Office and the Statistical Offices of the federal states (in Wiesbaden, Germany) on our behalf, based on our delivered analysis-syntaxes.

For our present analysis, we selected all inpatients hospitalized with PE (ICD code I26) (source: RDC of the Federal Statistical Office and the Statistical Offices of the federal states, DRG Statistics 2005–2020, own calculations). The PE patients were stratified according their CRB-65 score.

### 2.1. Study Endpoints and In-Hospital Adverse Events

The measured adverse in-hospital outcomes of this study were death of any cause during the hospital stay (in-hospital death), major adverse cardiac and cerebrovascular events (MACCE, including in-hospital death, myocardial infarction [ICD code I21] and/or ischaemic stroke [ICD code I63]), stroke (ischaemic or haemorrhagic) (ICD codes I61-I64), acute kidney injury (ICD code N17), and pneumonia (ICD-codes J12-J18), as well as serious bleeding events such as intracerebral bleeding (ICD code I61), gastrointestinal bleeding (ICD codes K920-K922), and necessity of transfusion of blood components (OPS code 8–800).

### 2.2. Definitions

Obesity was defined as a body mass index ≥30 kg/m^2^ as recommended by the World Health Organization (WHO). Stroke comprised both stroke entities: ischaemic and haemorrhagic stroke. Haemodynamically unstable PE was defined as PE patients with shock or cardiopulmonary resuscitation (CPR). Thrombophilia comprised antithrombin deficiency, protein C and S deficiency, prothrombin gene mutation, factor V Leiden mutation and other thrombophilia, including antiphospholipid (anticardiolipin) syndrome.

### 2.3. Ethical Aspects and Study Oversight

Since our study did not contain direct access by us investigators to data of individual patients, approval by an ethics committee and informed consent were not required, in compliance with German law.

### 2.4. Statistics

We computed the CRB-65 score (Table 1) and stratified the included PE patient cases for CRB-65 risk class: low-risk group (CRB-65 score 0 points) vs. high-risk group (CRB-65 score ≥1 points) [30,37].

Descriptive statistics for the comparison of both groups were provided with median and interquartile range (IQR), or absolute numbers and corresponding percentages; continuous variables were compared with the Wilcoxon–Whitney U test, whereas categorical variables were compared with Fisher’s exact or chi [2] test, as appropriate.

Logistic regression models were calculated to investigate associations between CRB-65 score as well as CRB-65 class (high-risk group [≥1 points] vs. low-risk group [0 points] as the reference) and adverse in-hospital events. In addition, the associations between calculated sPESI on the one side and case fatality, as well as MACCE on the other side, were calculated with logistic regressions. Results are presented as odds ratios (OR) and 95% confidence intervals (CI). To ensure and prove that the results of these logistic regressions are not substantially influenced by biasing factors (guaranteeing a wide independence of different cofactors), the multivariable logistic regressions were calculated with the following adjustments: age, sex, obesity, cancer, heart failure, essential arterial hypertension, hyperlipidaemia, acute and chronic kidney disease, diabetes mellitus, coronary artery disease, chronic obstructive pulmonary disease, atrial fibrillation/flutter, and pneumonia (only for the endpoint pneumonia—adjustment was adapted without pneumonia as an adjustment).

Temporal trends on total numbers of PE patients in the low-risk (0 points) and high-risk group (≥1 points) were analysed and the annual proportions of both groups were also illustrated.

To compare the prediction models of CRB-65 and sPESI, we calculated the receiver operating characteristic (ROC) curves with area under the curve (AUC) of CRB-65 as well as sPESI to predict case fatality and MACCE.

We used the software of SPSS^®^ (IBM Corp. Released 2011. IBM SPSS Statistics for Windows, Version 20.0. Armonk, NY: IBM Corp) for the computerized data analysis. Only P values of <0.05 (two-sided) were considered to be statistically significant.

## 3. Results

**Figure 1:**
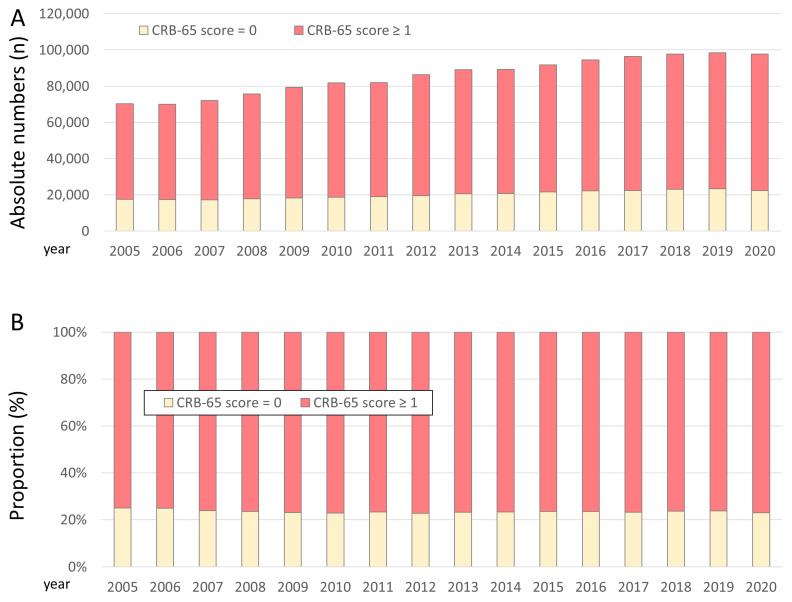
Time trends in PE patients stratified by CRB-65 score. (Panel (**A**)): annual time trends regarding absolute numbers of PE patients stratified by CRB-65 risk class during the observational period 2005–2020; (Panel (**B**)): annual time trends regarding proportions of PE patients of the two CRB-65 risk classes during the observational period 2005–2020.

Overall, 1,373,145 patient-cases of patients with PE with PE (76.6% aged ≥65 years, 47.0% females) were hospitalized in Germany between 2005 and 2020 and were included in our present study. As shown in Figure 1, the annual numbers of PE cases increased slowly from 2005 to 2020 (Figure 1A), whereas the proportion of high-risk patients according to the CRB-65 score (≥1 points) was widely stable over time (Figure 1B).

In total, 1,051,244 patient cases (76.6%) were classified as high-risk according to the CRB-65 score (≥1 points), while only 321,901 patient cases were categorized as low-risk according to the CRB-65 score (23.4%) (Table 2).

The majority of high-risk patients according to the CRB-65 score (≥1 points) were female (55.8%), while low-risk patients were more frequently male (56.4%). As expected, the in-hospital stay was longer in high-risk patients according to the CRB-65 score (≥1 points) in comparison to low-risk patients (10.0 [IQR 6.0–17.0] vs. 7.0 [4.0–12.0], *p* < 0.001). Besides obesity, all investigated cardiovascular risk factors were more prevalent in high-risk patients according to the CRB-65 score (≥1 points), resulting in an aggravated comorbidity profile in this patient group (Table 2). Consequently, the Charlson comorbidity index showed increased values in high-risk patients according to the CRB-65 score (≥1 points) in comparison to low-risk patients (5.0 [IQR 4.0–7.0] vs. 2.0 [0.0–3.0], *p* < 0.001).

Typical already-established risk stratification tools such as syncope (2.7% vs. 1.5%, *p* < 0.001) and right ventricular dysfunction (31.1% vs. 16.9%, *p* < 0.001), as well as tachycardia (3.2% vs. 2.2%, *p* < 0.001) and sPESI high-risk class (70.6% vs. 35.1%, *p* < 0.001) were all more present in the high-risk patients according to the CRB-65 score (≥1 points) (Table 2).

In-hospital death (19.0% vs. 3.4%, *p* < 0.001) and MACCE (22.4% vs. 5.1%, *p* < 0.001) occurred both distinctly more often in PE patients of the high-risk group according to the CRB-65 score (≥1 points) compared to the low-risk group (= 0 points) (Table 2). In addition, stroke, acute kidney injury, pneumonia, and all bleeding events were more present in the high-risk group according to the CRB-65 score (≥1 points).

Systemic thrombolysis (4.8% vs. 2.1%, *p* < 0.001) as well as surgical embolectomy (0.15% vs. 0.13%, *p* = 0.004) were more often used in the high-risk vs. low-risk group defined according to the CRB-65 score (Table 2).

Additionally, we analysed additionally the prognostic value of the high-risk group according to the CRB-65 score (≥1 points) for the prediction of adverse in-hospital events. The high-risk status according to the CRB-65 score (≥1 points) was independently related to a 3.81-fold increased risk of in-hospital death (OR 3.81 [95%CI 3.79–3.84], *p* < 0.001) and a 3.4-fold increased risk of MACCE (OR 3.35 [95%CI 3.32–3.37], *p* < 0.001) (Table 3). An increased CRB-65 score by 1 was also associated with all bleeding events, in particular those related to intracerebral bleeding (OR 2.35 [95%CI 2.29–2.42], *p* < 0.001), as well as the necessity of transfusion of blood constituents (OR 2.04 [95%CI 2.02–2.05], *p* < 0.001) (Table 3).

Furthermore, we consecutively analysed the prognostic value of the CRB-65 high-risk class for prediction of adverse in-hospital events. The CRB-65 high-risk class was independently and strongly associated with in-hospital death (OR 5.53 [95%CI 5.40–5.65], *p* < 0.001) as well as MACCE (OR 4.31 [95%CI 4.23–4.40], *p* < 0.001) (Table 4). Systemic thrombolysis (OR 5.39 [95%CI 5.23–5.55], *p* < 0.001) and surgical embolectomy (OR 3.15 [95%CI 2.79–3.56], *p* < 0.001) were associated with the CRB-65 high-risk group. The CRB-65 high-risk group was also independently associated with all bleeding events (Table 4).

**Figure 2:**
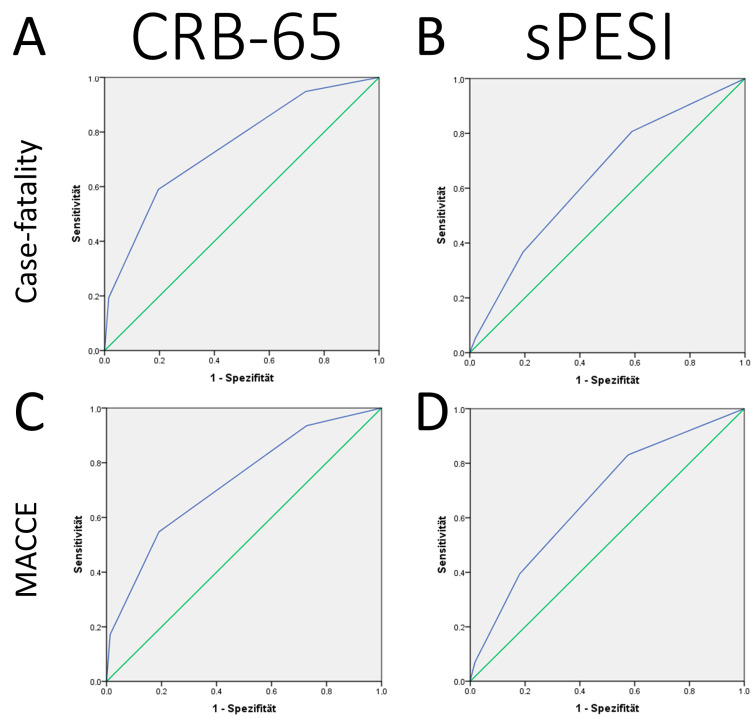
ROC curves for prediction of case fatality and MACCE. (Panel (**A**)): ROC curve for CRB-65 score to predict case fatality in PE patients during the observational period 2005–2020; (Panel (**B**)): ROC curve for sPESI to predict case fatality in PE patients during the observational period 2005–2020; (Panel (**C**)): ROC curve for CRB-65 score to predict MACCE in PE patients during the observational period 2005–2020; (Panel (**D**)): ROC curve for sPESI to predict MACCE in PE patients during the observational period 2005–2020.

We compared the prognostic performance of the CRB-65 score with the established sPESI with the help of ROC curves. The AUC for the prediction of case fatality as well as MACCE of CRB-65 was better in comparison to the sPESI; while the CRB-65 score revealed an AUC of >0.7 (AUC 0.746 [95%CI 0.744–0.747], *p* < 0.001) for prediction of case fatality, sPESI showed an AUC <0.7 (AUC 0.640 [95%CI 0.639–0.642], *p* < 0.001). Similarly, the AUC of CRB-65 to predict MACCE was higher (AUC 0.727 [95%CI 0.726–0.728], *p* < 0.001) than that of sPESI for the prediction of MACCE (AUC 0.669 [95%CI 0.66–0.670], *p* < 0.001) (Figure 2).

The sPESI high-risk class was independently associated with case fatality (univariably logistic regression: OR 2.92 [95%CI 2.88–2.95], *p* < 0.001; multivariable logistic regression: OR 1.73 [95%CI 1.71–1.76], *p* < 0.001) as well as MACCE (univariably logistic regression: OR 3.61 [95%CI 3.57–3.65, *p* < 0.001; multivariable logistic regression: OR 2.50 [95%CI 2.46–2.55], *p* < 0.001).

## 4. Discussion

PE is accompanied by high morbidity and mortality [1,2,3,4,5,6]. The mortality of PE events is closely related to haemodynamic status, cardiac deviations with RVD and/or myocardial injury, as well as comorbidity profile [2,3,4,6,7,8,9,10,11,12,13]. Besides these risk stratification parameters, several scores have been developed and established and are already in use for the prediction of PE prognosis [16,17,18,19,20,21,22,23,24,25] Nevertheless, for clinical routines, simpler, faster and more easily assessable scores with favourable effectiveness are wanted [6,18].

The main results of our present study can be summarized as follows:

(i) Annual numbers of PE cases increased slowly from 2005 to 2020.
(ii) The proportion of high-risk patients according to the CRB-65 score (≥1 points) was widely stable over time.
(iii) Established risk stratification parameters such as syncope and right ventricular dysfunction, as well as tachycardia and sPESI, were more prevalent in the high-risk patients according to the CRB-65 score (≥1 points).
(iv) In-hospital case fatality rate was 15.6%, and MACCE rate 17.3% higher in PE patients of the high-risk group according to the CRB-65 score (≥1 points) compared to the low-risk group (= 0 points). In addition, stroke, acute kidney injury, pneumonia, and all bleeding events occurred more often in the high-risk group according to the CRB-65 score (≥1 points).
(v) Systemic thrombolysis as well as surgical embolectomy were both more often used in the high-risk vs. low-risk group defined according to the CRB-65 score.
(vi) An increase in CRB-65 score by 1 was independently related to a 3.8-fold higher risk for in-hospital death and a 3.4-fold higher risk for MACCE.
(vii) The CRB-65 high-risk class was independently and strongly associated with in-hospital death as well as MACCE.
(viii) The prognostic performance of the CRB-65 score was better as sPESI, wherby the sPESI was developed for risk stratification of haemodynamically stable PE patients.
(ix) Systemic thrombolysis and surgical embolectomy were both independently more often used in the CRB-65 high-risk group.
(x) The CRB-65 high-risk group was also independently associated with all bleeding events.

Thus, the CRB-65 score shows an acceptable prognostic performance to identify PE patients, who are at higher risk of dying during the initial phase of the PE event. In addition, the CRB-65 score was able to predict other adverse in-hospital events, including bleeding. These results are in accordance with one small previously published study, which demonstrated that the CRB-65 score might also be useful for risk stratification in patients with acute PE [18].

For PE, several scores are established to predict short-term outcomes (especially survival) comprising the PESI, sPESI, and other scores, which are already in use for prediction of PE patients’ prognosis [6,16,17,18,19,20,21,22,23,24,25]. In addition, several scores are designed to predict the bleeding risk of PE patients [6,38,39,40,41,42]. Nevertheless, most of these scores are not simple and easy to access, since several different and more complex parameters have to be considered. Additionally, the aforementioned scores are specialized for PE and lack generalization regarding prognosis evaluation of other pulmonary diseases [18]. This might be a disadvantage in the early triage/management of patients with acute dyspnoea without having established the PE diagnosis. In this context, patients with pneumonia can present at the emergency departments with similar symptoms to those caused by PE [43,44].

In patients with pneumonia, CRB-65 is well validated and is widely used for severity assessment and initial risk-adapted management of patients with pneumonia [29,30,31,32,33,34]. The key advantage of CRB-65 is that this score can easily be assessed based on the criteria of new-onset confusion, respiratory failure (respiratory rate ≥30 /min), haemodynamic compromise/shock/CPR (systolic blood pressure <90 mmHg or diastolic blood pressure ≤60 mmHg), and age ≥65 years [29,31,33]. Since the haemodynamic status with the identification of haemodynamic compromise is also one of the key aspects of good and efficiently working ESC risk stratification [6], it is not a surprise that the prognostic value of the CRB-65 score is at least acceptable. The ESC risk stratification additively uses the sPESI, PESI, RVD (diagnosed at CT or at the echocardiography) and cardiac troponins for further risk assessment of haemodynamically stable PE patients [6]. Of course, it is to be expected that the ESC guideline-based risk stratification approach be far more precise for the risk stratification of PE patients, but besides the haemodynamical compromise, these parameters of the ESC guidelines are not easily accessible, and their results are not available in the first short minutes after the arrival of the patients at the emergency department. In addition, the PE diagnosis is at this time point not already established [6]. Thus, the CRB-65 score might be an ideal initial risk stratification approach regarding patients with acute dyspnoea regardless of an underlying pneumonia vs. PE in these patients (before a definite diagnosis is made) in order to distinguish between patients who are at high risk of death from those with lower risk of dying during the first hours after admission with the requirement of being monitored and managed at an intensive care unit. In this context, it has to be mentioned that our study did not show an inferiority of the CRB-65 score to predict case fatality as well as MACCE in comparison to the sPESI in this crucial patient group, wherby the sPESI was developed for risk stratification of haemodynamically stable patients and we investigated the prognostic performance of both tools (CRB-65 as well as sPESI) in all hospitalized PE patients regardless of haemodynamic status.

Since the score is based exclusively on clinical, immediately assessable variables, which should anyhow be assessed in the initial work-up after the admission of these patients with dyspnoea, there is only a very small delay by calculating the CRB-65 score without additional technical expense [30,31,33,35].

Thus, in summary, CRB-65 seems to be a promising and simple risk stratification approach/tool to identify PE patients at higher risk of in-hospital death and adverse in-hospital events, especially before establishing the definitive diagnosis of PE or pneumonia in patients with acute dyspnoea.

## 5. Limitations

Several limitations of our present study merit consideration: First, as our results are based on administrative coding data, we cannot exclude misclassification or inconsistencies. In this context, our analysis of the German nationwide inpatient sample was not prespecified, and therefore, the findings of the study can only be considered to be hypothesis-generating. Second, patients with PE who died out of hospital were not included in the German nationwide inpatient sample. Third, the German nationwide inpatient sample does not report follow-up outcomes after discharge from hospital. Fourth, due to coding reasons, we were not able to present D-Dimer and antithrombin levels as well as further echocardiographic parameters, in detail. Fifth, anticoagulation treatment is not coded and assessable in the German nationwide sample.

## 6. Conclusions

Risk stratification with CRB-65 score was helpful for identifying PE patients at higher risk of adverse in-hospital events. The high-risk class according to CRB-65 score (≥1 point) was independently associated with a striking 5.5-fold occurrence of in-hospital death. CRB-65 seems to be a promising and simple risk stratification approach/tool to identify PE patients at higher risk of in-hospital death and adverse in-hospital events, and might be similarly predictive to sPESI, especially before establishing the definitive diagnosis of PE or pneumonia in patients with acute dyspnoea.

## Figures and Tables

**Table 1:** Items of the CRB-65 score and analysed diagnoses.

	CRB-65 Point Score	ICD or OPS Codes
Confusion	+1 point	ICD code R40
Respiratory failure	+1 point	ICD code J96 and/or OPS codes 8–71 or 8–72
Unstable pulmonary embolism (CPR or shock)	+1 point	ICD code R57 and/or OPS code 8–77
Age ≥65 years	+1 point	
Graduation of patients according to CRB-65 score: Low-risk group: 0 points High-risk group: ≥1 points		

**Table 2:** Patients’ characteristics, medical history, presentation, and outcomes of the included 1,373,145 patients with pulmonary embolism stratified according to the CRB-65 score.

Parameters	PE Patients with CRB-65 Score = 0 (*n* = 321,901; 23.4%)	PE Patients with CRB-65 Score ≥ 1 (*n* = 1,051,244; 76.6%)	*p*-Value
Age	53.0 (44.0–59.0)	76.0 (69.0–82.0)	<0.001
Age ≥65 years	0 (0.0%)	921,165 (87.6%)	<0.001
Female sex *	140,497 (43.6%)	586,987 (55.8%)	<0.001
In-hospital stay (days)	7.0 (4.0–12.0)	10.0 (6.0–17.0)	<0.001
**Traditional cardiovascular risk factors**			
Obesity	35,267 (11.0%)	95,375 (9.1%)	<0.001
Essential arterial hypertension	91,902 (28.5%)	510,857 (48.6%)	<0.001
Diabetes mellitus	30,219 (9.4%)	226,017 (21.5%)	<0.001
Hyperlipidaemia	24,257 (7.5%)	147,887 (14.1%)	<0.001
**Classical risk factors for venous thromboembolism and proportion of DVT**			
Cancer	68,567 (21.3%)	210,606 (20.0%)	<0.001
Any surgery	154,930 (48.1%)	556,953 (53.0%)	<0.001
Thrombophilia	7853 (2.4%)	8218 (0.8%)	<0.001
Deep venous thrombosis or thrombophlebitis	138,595 (43.1%)	350,439 (33.3%)	<0.001
**Comorbidities**			
Charlson comorbidity index	2.0 (0.0–3.0)	5.0 (4.0–7.0)	<0.001
Coronary artery disease	16,262 (5.1%)	171,331 (16.3%)	<0.001
Heart failure	24,245 (7.5%)	276,552 (26.3%)	<0.001
Peripheral artery disease	4092 (1.3%)	35,586 (3.4%)	<0.001
Atrial fibrillation/flutter	12,069 (3.7%)	194,995 (18.5%)	<0.001
Chronic obstructive pulmonary disease	14,190 (4.4%)	124,215 (11.8%)	<0.001
Acute and chronic kidney disease	19,514 (6.1%)	274,962 (26.2%)	<0.001
**Risk stratification markers of VTE**			
Unstable PE (CPR or shock)	0 (0.0%)	123,180 (11.7%)	<0.001
Shock	0 (0.0%)	56,644 (5.4%)	<0.001
Syncope	4673 (1.5%)	28,643 (2.7%)	<0.001
Right ventricular dysfunction	54,433 (16.9%)	326,828 (31.1%)	<0.001
Tachycardia	6955 (2.2%)	33,964 (3.2%)	<0.001
Respiratory failure	0 (0.0%)	394,858 (37.6%)	<0.001
Confusion	0 (0.0%)	25,385 (2.4%)	<0.001
sPESI ≥1 (sPESI high-risk class)	113,092 (35.1%)	741,874 (70.6%)	<0.001
**Adverse events during hospitalization**			
In-hospital death	10,874 (3.4%)	199,702 (19.0%)	<0.001
MACCE	16,318 (5.1%)	235,908 (22.4%)	<0.001
Systemic thrombolysis	6645 (2.1%)	50,532 (4.8%)	<0.001
Surgical embolectomy	417 (0.13%)	1593 (0.15%)	0.004
Pneumonia	73,215 (22.7%)	257,620 (24.5%)	<0.001
Acute kidney injury	5423 (1.7%)	84,936 (8.1%)	<0.001
Stroke (ischaemic or haemorrhagic)	4816 (1.5%)	35,764 (3.4%)	<0.001
Intracerebral bleeding	1034 (0.3%)	7531 (0.7%)	<0.001
Gastrointestinal bleeding	2335 (0.7%)	18,594 (1.8%)	<0.001
Transfusion of blood constituents	21,462 (6.7%)	138,194 (13.1%)	<0.001

* Information available for 1,373,084 patients.

**Table 3:** Prognostic value of the CRB-65 score for prediction of adverse in-hospital events. Association of an increase in CRB-65 score by 1 with the adverse in-hospital events (univariable and multivariable logistic regression model).

	Univariable Regression Model		Multivariable Regression Model *	
	OR (95% CI)	*p*-Value	OR (95% CI)	*p*-Value
In-hospital death	3.72 (3.69–3.74)	<0.001	3.81 (3.79–3.84)	<0.001
MACCE	3.37 (3.35–3.39)	<0.001	3.35 (3.32–3.37)	<0.001
Pneumonia	1.28 (1.27–1.28)	<0.001	1.45 (1.44–1.45)	<0.001
Acute kidney injury	2.96 (2.94–2.99)	<0.001	2.37 (2.34–2.39)	<0.001
Stroke (ischaemic or haemorrhagic)	1.70 (1.69–1.72)	<0.001	1.77 (1.74–1.79)	<0.001
Intracerebral bleeding	1.89 (1.85–1.94)	<0.001	2.35 (2.29–2.42)	<0.001
Gastrointestinal bleeding	1.72 (1.69–1.75)	<0.001	1.51 (1.48–1.54)	<0.001
Transfusion of blood constituents	1.89 (1.87–1.90)	<0.001	2.04 (2.02–2.05)	<0.001

* Adjusted for age, sex, obesity, cancer, heart failure, essential arterial hypertension, hyperlipidemia, acute and chronic kidney disease, diabetes mellitus, coronary artery disease, chronic obstructive pulmonary disease, atrial fibrillation/flutter, and pneumonia (only for the endpoint pneumonia—adjustment was adapted without pneumonia as an adjustment).

**Table 4:** Prognostic value of the CRB-65 high-risk class for prediction of adverse in-hospital events. Association of high-risk class with the adverse in-hospital events (univariable and multivariable logistic regression model).

	Univariable Regression Model		Multivariable Regression Model *	
	OR (95% CI)	*p*-Value	OR (95% CI)	*p*-Value
In-hospital death	6.71 (6.58–6.84)	<0.001	5.53 (5.40–5.65)	<0.001
MACCE	5.42 (5.33–5.51)	<0.001	4.31 (4.23–4.40)	<0.001
Right ventricular dysfunction	2.22 (2.20–2.24)	<0.001	2.42 (2.39–2.45)	<0.001
Systemic thrombolysis	2.40 (2.33–2.46)	<0.001	5.39 (5.23–5.55)	<0.001
Surgical embolectomy	1.17 (1.05–1.30)	<0.001	3.15 (2.79–3.56)	<0.001
Pneumonia	1.10 (1.09–1.11)	<0.001	1.49 (1.47–1.51)	<0.001
Acute kidney injury	5.13 (4.99–5.27)	<0.001	2.97 (2.86–3.09)	<0.001
Stroke (ischaemic or haemorrhagic)	2.32 (2.25–2.39)	<0.001	2.49 (2.40–2.58)	<0.001
Intracerebral bleeding	2.24 (2.10–2.39)	<0.001	3.97 (3.68–4.28)	<0.001
Gastrointestinal bleeding	2.46 (2.36–2.57)	<0.001	1.89 (1.79–1.99)	<0.001
Transfusion of blood constituents	2.12 (2.09–2.15)	<0.001	2.75 (2.70–2.81)	<0.001

* Adjusted for age, sex, obesity, cancer, heart failure, essential arterial hypertension, hyperlipidemia, acute and chronic kidney disease, diabetes mellitus, coronary artery disease, chronic obstructive pulmonary disease, atrial fibrillation/flutter, and pneumonia (only for the endpoint pneumonia—adjustment was adapted without pneumonia as an adjustment).

## Data Availability

Statistical analyses were performed on our behalf by the Research Data Center (RDC) of the Federal Bureau of Statistics (Wiesbaden, Germany) (source: RDC of the Federal Statistical Office and the Statistical Offices of the federal states, DRG Statistics 2005–2020, and own calculations).
